# Predicting emergency department visits in a large teaching hospital

**DOI:** 10.1186/s12245-021-00357-6

**Published:** 2021-06-12

**Authors:** Nathan Singh Erkamp, Dirk Hendrikus van Dalen, Esther de Vries

**Affiliations:** 1 TiSEM, Tilburg School of Economics and Management, Tilburg University, PO Box 90153, 5000LE Tilburg, the Netherlands; 2 Jeroen Bosch Academy Research, Jeroen Bosch Hospital, PO Box 90153, 5200ME ’s-Hertogenbosch, the Netherlands; 3 Tilburg School of Social and Behavioral Sciences, Tilburg University, 5000LE Tranzo, Tilburg, the Netherlands

**Keywords:** Emergency department visits, Prediction, Weather, Calendar data

## Abstract

**Background:**

Emergency department (ED) visits show a high volatility over time. Therefore, EDs are likely to be crowded at peak-volume moments. ED crowding is a widely reported problem with negative consequences for patients as well as staff. Previous studies on the predictive value of weather variables on ED visits show conflicting results. Also, no such studies were performed in the Netherlands. Therefore, we evaluated prediction models for the number of ED visits in our large the Netherlands teaching hospital based on calendar and weather variables as potential predictors.

**Methods:**

Data on all ED visits from June 2016 until December 31, 2019, were extracted. The 2016–2018 data were used as training set, the 2019 data as test set. Weather data were extracted from three publicly available datasets from the Royal Netherlands Meteorological Institute. Weather observations in proximity of the hospital were used to predict the weather in the hospital’s catchment area by applying the inverse distance weighting interpolation method. The predictability of daily ED visits was examined by creating linear prediction models using stepwise selection; the mean absolute percentage error (MAPE) was used as measurement of fit.

**Results:**

The number of daily ED visits shows a positive time trend and a large impact of calendar events (higher on Mondays and Fridays, lower on Saturdays and Sundays, higher at special times such as carnival, lower in holidays falling on Monday through Saturday, and summer vacation). The weather itself was a better predictor than weather volatility, but only showed a small effect; the calendar-only prediction model had very similar coefficients to the calendar+weather model for the days of the week, time trend, and special time periods (both MAPE’s were 8.7%).

**Conclusions:**

Because of this similar performance, and the inaccuracy caused by weather forecasts, we decided the calendar-only model would be most useful in our hospital; it can probably be transferred for use in EDs of the same size and in a similar region. However, the variability in ED visits is considerable. Therefore, one should always anticipate potential unforeseen spikes and dips in ED visits that are not shown by the model.

**Supplementary Information:**

The online version contains supplementary material available at 10.1186/s12245-021-00357-6.

## Background

Large numbers of patients generally present at emergency departments (EDs). In the Netherlands, 2.3 million patients were seen on EDs in 2017 [1]. ED visits show a high volatility over time [2–4]. Historically, many EDs have been staffed based on average patient volumes [5], resulting in EDs that are more likely to be crowded at peak-volume moments.

ED crowding is defined as a situation in which the demand for emergency services exceeds the ability of the department to provide quality care within acceptable time frames [6]. ED crowding is a widely reported problem with negative consequences for patients as well as staff. Two large systematic reviews report various negative consequences for patients, including treatment delay and increased mortality [7, 8], increased frequency of exposure to error, increased risk of readmission, and reduced patient satisfaction [8]. Reported consequences for staff include higher stress levels, increased violence towards staff, and inability to adhere to guideline-recommended treatment [8].

Both these systematic reviews included articles that identified insufficient staffing as a possible cause for ED crowding, and additional staffing as a possible solution [7, 8]. To ensure adequate staffing only when needed, a flexible, volume-based staffing plan could be considered [5, 9]. This can be achieved by analyzing available patient arrival patterns followed by developing a predictive model [5, 10]. Possibly relevant predictors of patient arrival patterns are calendar data [2] and weather variables [11–13].

The predictive value of weather variables on ED visits is not yet clear as previous studies show conflicting results. Climatic differences between countries may be a possible explanation for this. Also, population’s adaptations to local climatic circumstances might be of influence [14, 15]. Furthermore, holidays may differ between countries and cultures, as they often have a national or cultural character. As far as we know, no previous prediction models using weather and calendar data have been developed for hospitals in the Netherlands. Therefore, the aim of this study was to create a prediction model for the number of ED visits in the Jeroen Bosch Hospital as a representative example, based on calendar data and weather variables as potential predictors.

## Methods

### Emergency department visits

Data on all ED visits in the Jeroen Bosch Hospital, a large teaching hospital in the Netherlands, from June 2016 until December 31, 2019, were extracted and anonymized. The data set contained the birth year and gender of the visitor and the date and time of admission to the ED. The 2016–2018 data were used as training set, the 2019 data as test set.

### Calendar variables

**Fig. 1:**
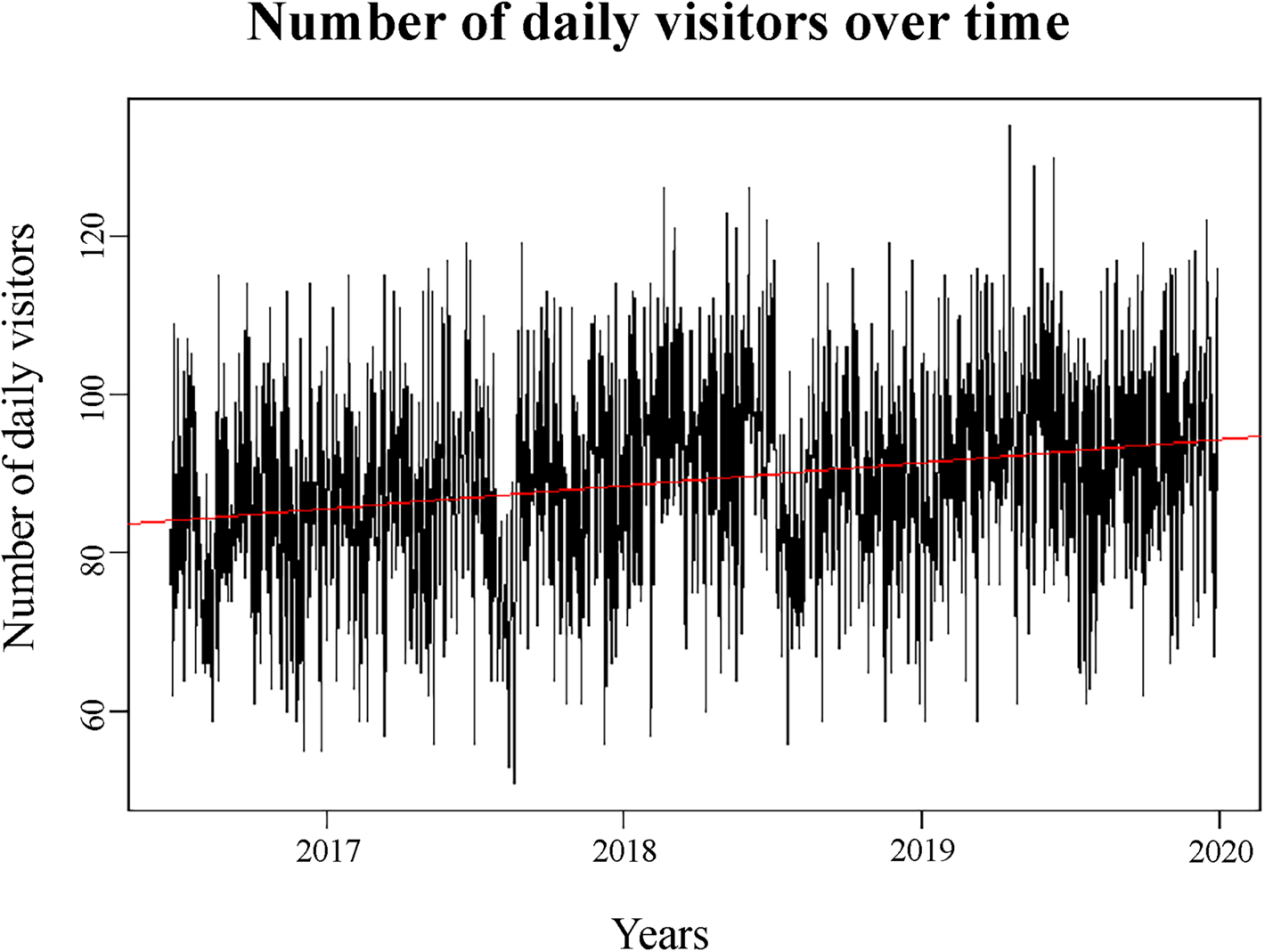
Emergency department visits in the Jeroen Bosch Hospital over time

**Fig. 2:**
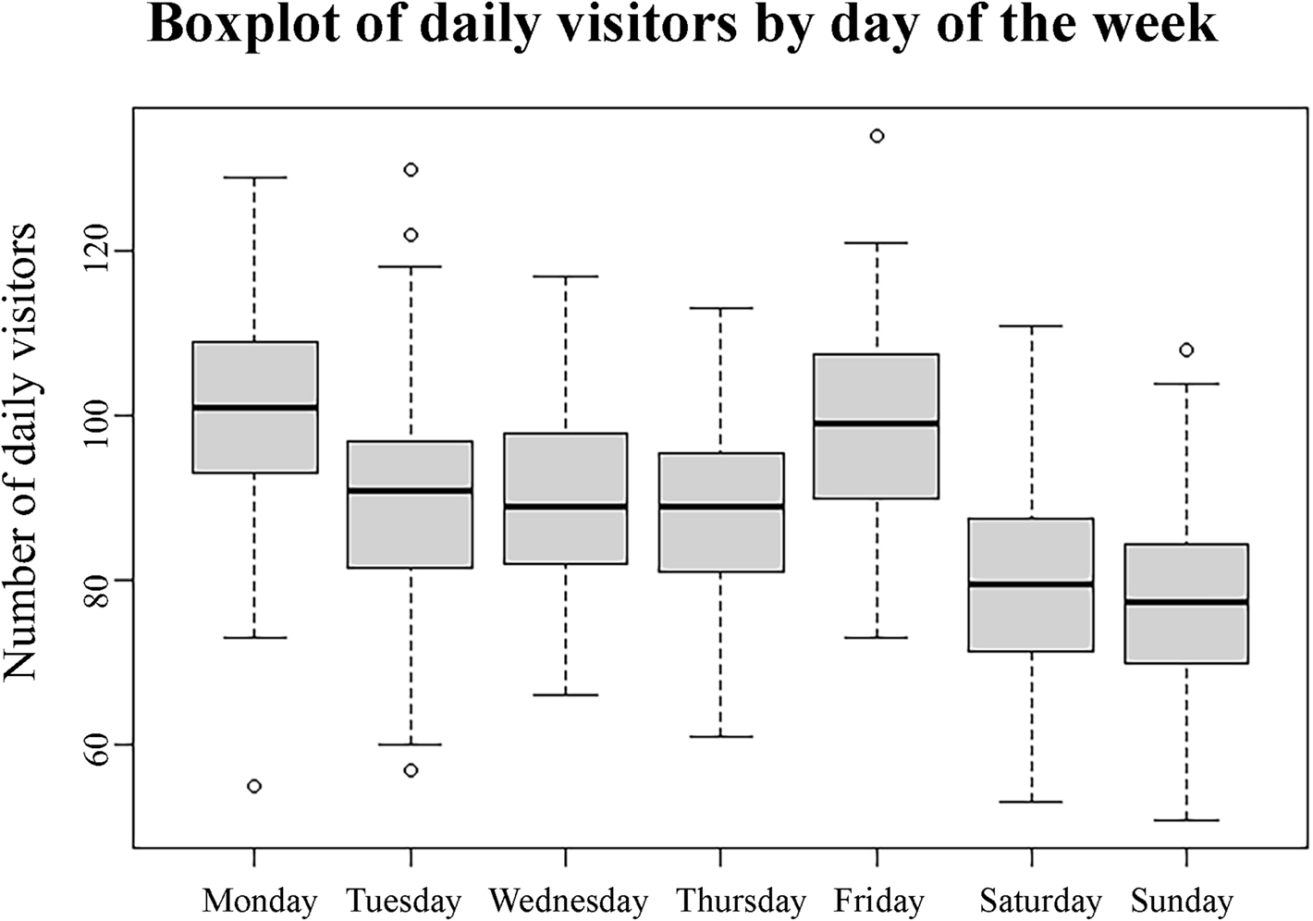
Emergency department visits in the Jeroen Bosch Hospital by day of the week

**Fig. 3:**
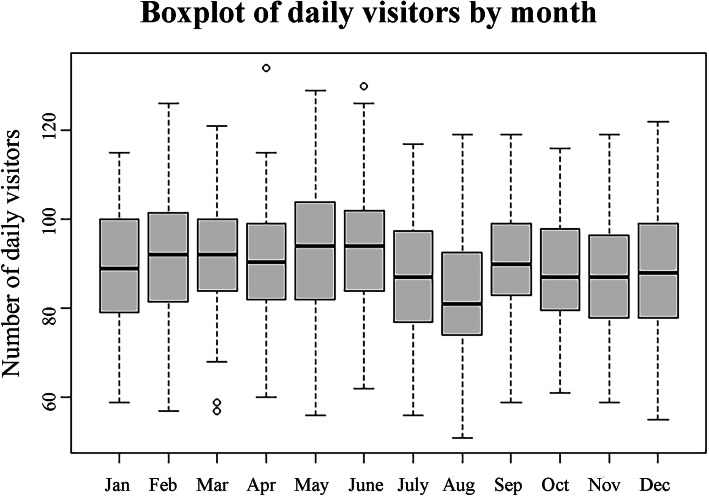
Emergency department visits in the Jeroen Bosch Hospital by calendar month. Jan January, Feb February, Mar March, Apr April, Aug August, Sep September, Oct October, Nov November, Dec December

Descriptive analysis of the ED visit data showed a positive time trend (Fig. 1), and influence of the day of the week (Fig. 2), month (Fig. 3), and summer vacation and holidays on the number of daily ED visits (details in additional file 1). All these were added as potential predictors to the models.

### Weather data

Weather data were extracted from three publicly available datasets from the Royal Netherlands Meteorological Institute (KNMI): daily readings from the automatic weather stations [16], hourly readings from the automatic weather stations [17], and daily readings from the precipitation stations [18]. The daily readings from the automatic weather stations provide an extensive characterization of the observed wind speed, temperature, radiation, pressure, visibility, cloudiness, humidity, and precipitation in the Netherlands; the daily readings from the precipitation stations and the hourly readings from the automatic weather stations further outline the occurred precipitation as well as special weather conditions such as fog, glazed frost, and storms (details in additional file 2).

**Fig. 4:**
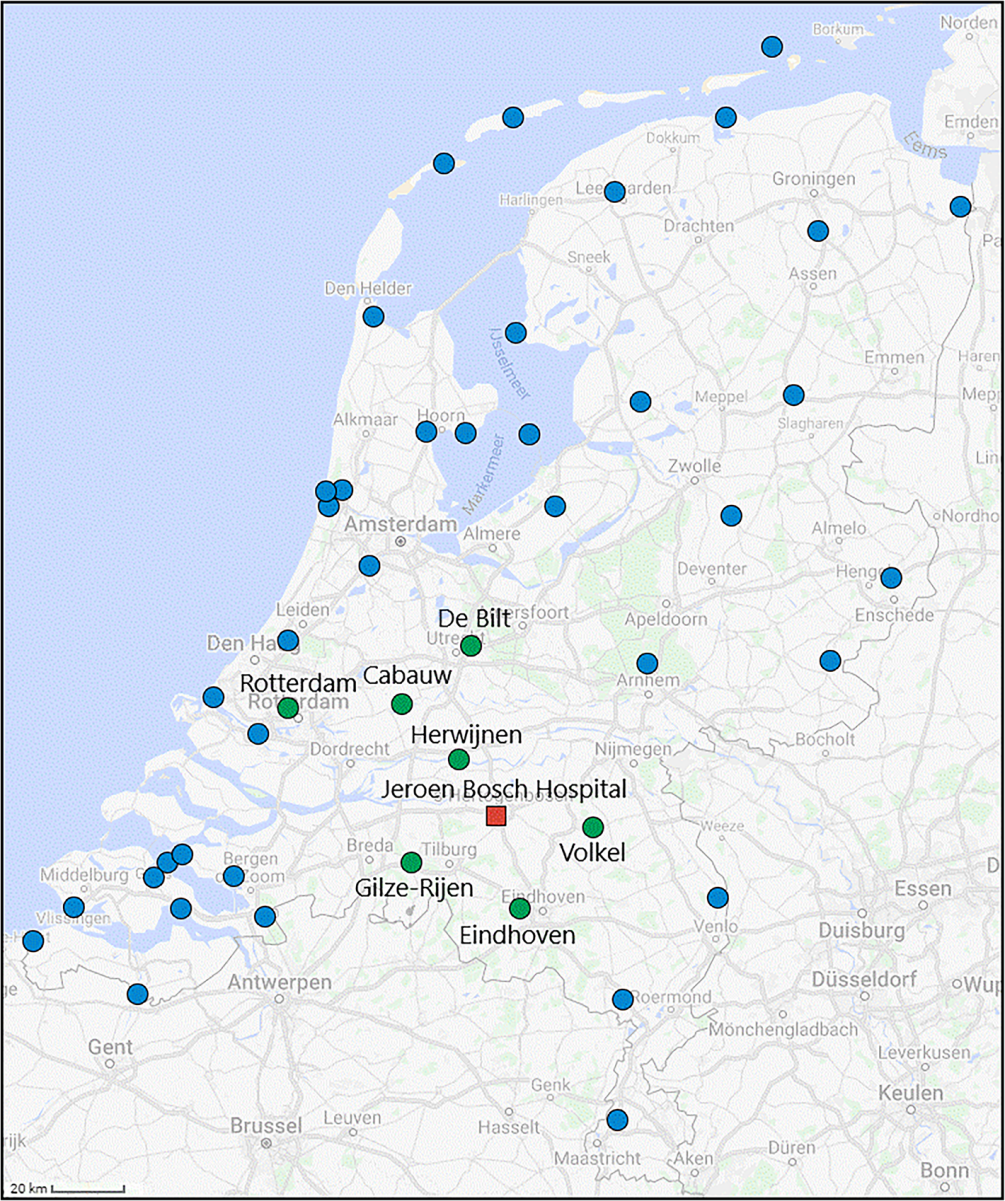
The location of the KNMI automatic weather stations. Red square = the Jeroen Bosch Hospital, green dots = selected automatic weather stations, blue dots = not selected automatic weather stations

As shown in Fig. 4, there are no weather stations in close proximity of the Jeroen Bosch Hospital, and only a few are located not too far away. The weather observations from the KNMI weather stations in proximity of the Jeroen Bosch Hospital (green dots in Fig. 4) were used to predict the weather in the Jeroen Bosch Hospital’s catchment area by applying the inverse distance weighting interpolation method [19]. The parameter of this interpolation method is estimated using leave-one-out cross-validation, where only the KNMI weather stations within the catchment area of the Jeroen Bosch Hospital are left out one at a time (details in additional file 3).

Two sets of weather-related predictors were created by applying the inverse distance weighting weather interpolation method, based on two alternative hypotheses. The first set contained the weather data predicted at the Jeroen Bosch Hospital as a means to describe the weather experienced by ED visitors. The second set contained the weather data predicted for two towns in the periphery of the Jeroen Bosch Hospital’s catchment area, Kaathoven and Drunen. The use of weather predictions at these locations has the potential to better describe the weather in the full catchment area than weather predictions at one central location (the Jeroen Bosch Hospital).

### Prediction modeling

The predictability of daily ED visits was examined by creating linear prediction models. As many of the weather predictors were likely not to have a substantial effect on the number of ED visits, a variable selection method was used to refine and merge the available sets of calendar and weather-based predictors. This was done using the stepwise selection method which, starting with an empty model, adds or drops predictors one at a time in order to maximize improvement in some measurement of fit. For the measurement of fit the Akaike information criterion (AIC) was used, which estimates the out-of-sample error using only the sum of squared residuals of the model, the number of predictors in the model, and the number of observations in the training set. This variable selection method was applied on both the set of calendar variables plus the first set of weather-related predictors and on the set of calendar variables plus the second set of weather-related predictors in order to find suitable ED visit predictors. The results were used to evaluate the quality of predictors and refine/merge the sets of predictors accordingly. The final daily ED visits’ linear prediction model based on calendar plus weather-based predictors was created by reapplying stepwise selection on this refined set of predictors.

The effect of predicted weather on the number of ED visits was evaluated by comparing the accuracy of test set predictions from the calendar and weather-based model with the accuracy of a linear prediction model without the weather variables from the final prediction model based on calendar plus weather-based predictors. The mean absolute percentage error (MAPE) was used as measurement of fit for the predictions, which allowed for interpretation as the average percentage the predictions are off compared to the true number of daily ED visits and for comparisons with ED visits models of other hospitals. The difference in MAPE of the models was used to verify whether and how much the weather affects the number of daily ED visits at the Jeroen Bosch Hospital.

## Results

### Variable selection

Automated stepwise variable selection on dataset A (calendar- plus Jeroen Bosch Hospital-weather-based predictors; Table 1) showed that most variables present in dataset A were relevant for predicting daily ED visits, with the majority of the missing calendar-based variables being indicators for months. A small set of interaction variables was selected by stepwise selection as well. Automated stepwise variable selection on dataset B (calendar- plus Kaathoven/Drunen-weather-based predictors; Table 2) showed similar results, with generally one of these cities having a positive coefficient and one having an equally sized negative coefficient, illustrating that the volatility of the weather could also be a meaningful predictor of daily ED visits.

### Merging and refining the sets of predictors

Dataset C (the refined set of predictors) is created by combining the best predictors found in datasets A and B using stepwise selection. This new set of predictors contains most calendar-based predictors from the previous sets, with the seasonal indicators replacing the monthly indicators which had a small effect on ED visits and were often omitted by stepwise selection. A small selection of important interactions seen in the results of the stepwise selections was included as well. For the description of the weather, 24 predictors were included, 12 weather variables describing 12 weather phenomena using weather predictions at the Jeroen Bosch Hospital and 12 weather variables describing the volatility of these 12 weather phenomena using the absolute difference in predicted weather between Kaathoven and Drunen. A more extensive description of the predictors in the new set can be found in additional file 4.

### Weather- and calendar-based prediction model

The final calendar- and weather-based daily ED visits linear prediction model is created by reapplying stepwise selection on dataset C (Table 3) and shows higher average daily ED visits on Mondays and Fridays as well as lower average daily ED visits on Saturday and Sunday. The number of daily ED visits is also affected by a positive time trend and special time periods such as carnival, holidays falling on Monday through Saturday, and summer vacation. A selection of weather predictions at the Jeroen Bosch Hospital was included by stepwise selection as well, illustrating that the weather itself made for better daily ED visits predictors than the volatility of the weather.

### Calendar-only model

The calendar-only dataset D that is used to assess the importance of weather for predicting ED visits consists of the calendar variables selected from dataset C in stepwise selection plus the seasonal indicators omitted from this model. The calendar-only prediction model (Table 4) created using all predictors from this dataset has very similar coefficients for the days of the week, time trend and special time periods as the model created by applying stepwise selection on dataset C (Table 3). The effects of the calendar variables on the predicted number of ED visits are shown in Table 5.The prediction performance of these two models turned out to be very similar (Table 6), both on average making predictions that are 8.7% off compared to the true number of daily ED visits.

**Table 1:** Stepwise variable selection on dataset A

Variable	Estimate	Standard error	t-value	*P*-value	Significance level
(Intercept)	248.7786337	45.4299646	5.476	5.67e−08	<.001
Monday	11.5596282	1.0533831	10.974	<2e−16	<.001
Friday	8.4939099	1.1411012	7.444	2.32e−13	<.001
Saturday	− 8.3012065	1.1525279	− 7.203	1.26e−12	<.001
Sunday	− 10.3104139	2.0117598	-5.125	3.65e−07	<.001
January	2.3137231	1.4929930	1.550	0.121566	N.S.
May	− 3.2596707	1.4369325	− 2.268	0.023540	<.05
August	− 3.3159900	1.3688478	− 2.422	0.015616	<.05
Friday * Spring	6.7473582	2.2399643	3.012	0.002667	<0.01
Sunday * Summer	3.4651415	2.0302316	1.707	0.088216	N.S.
Tuesday * Fall	− 4.7872202	1.7387084	− 2.753	0.006020	<0.01
Thursday * Fall	− 4.4414934	1.7371769	− 2.557	0.010732	<.05
Saturday * Winter	− 5.2470915	2.2009207	− 2.384	0.017334	<.05
Summer vacation week 1 + 2	− 8.0769673	1.7847806	− 4.525	6.85e−06	<.001
Summer vacation week 3 + 4	− 10.5916099	1.8959655	− 5.586	3.09e−08	<.001
Thursday * Summer vacation	3.5492285	2.5010552	1.419	0.156225	N.S.
Friday * Vacation	− 6.5440714	2.9636941	− 2.208	0.027495	<.05
Holiday * Vacation	− 5.0010994	3.2588828	− 1.535	0.125239	N.S.
Monday * Holiday	− 21.0261795	3.6481167	− 5.764	1.14e−08	<.001
Thursday * Holiday	− 21.0240305	5.8145506	− 3.616	0.000316	<.001
Wednesday * Holiday	− 19.0692120	7.1177032	− 2.679	0.007519	<0.01
Friday * Holiday	− 27.1374017	9.8894943	− 2.744	0.006191	<0.01
Carnival	10.7865927	4.1303939	2.612	0.009167	<0.01
Time trend	0.0097392	0.0013999	6.957	6.77e−12	<.001
Sunday * Time trend	− 0.0053839	0.0034610	− 1.557	0.120165	N.S.
Summer vacation * Time trend	− 0.0125371	0.0026263	− 4.772	2.12e−06	<.001
Maximum Temperature	0.0360680	0.0088407	4.080	4.92e−05	<.001
Winter * Mean temperature	− 0.0568999	0.0255135	− 2.527	0.011666	<.05
Radiation	0.0029971	0.0009226	3.249	0.001203	<0.01
Maximum pressure	− 0.0155889	0.0044522	− 3.501	0.000486	<.001
Winter * Maximum pressure	0.0007034	0.0001660	4.238	2.49e−05	<.001
Precipitation duration	0.0407058	0.0162960	2.498	0.012674	<.05
Summer * Precipitation (automatic stations)	− 0.312219	0.0081872	− 3.814	0.000147	<.001
Maximum humidity	− 0.1241583	0.0719627	− 1.725	0.084820	N.S.
Maximum visibility	− 0.0086327	0.0042522	− 2.030	0.042636	<.05
Hours of fog	0.4447461	0.1570946	2.831	0.004744	<.01
Winter * hours of snow	− 1.2095289	0.3781155	− 3.199	0.001429	<.01
Residual standard error	9.668 on 884 degrees of freedom				
Multiple R-squared	0.5307				
Adjusted R-squared	0.5115				
F-statistic	27.76 on 36 and 884 degrees of freedom, *P*-value: < 2.2e−16				

Dataset A is based on the calendar variables plus weather predictions at the Jeroen Bosch Hospital; * = interaction; *N.S.* not significant

**Table 2:** Stepwise variable selection on dataset B

Variable	Estimate	Standard error	t-value	*P*-value	Significance level
(Intercept)	248.911211	46.168830	5.391	9.01e−08	<.001
Monday	11.186836	1.049152	10.663	<2e−16	<.001
Friday	9.124763	1.405000	6.494	1.40e−10	<.001
Saturday	− 8.856937	1.151790	− 7.690	3.98e−14	<.001
Sunday	− 9.348118	1.858019	− 5.031	5.92e−07	<.001
May	− 2.833858	1.505424	− 1.882	0.060110	N.S.
August	− 2.783141	1.467921	− 1.896	0.058294	N.S.
Friday * Spring	6.425346	2.359589	2.723	0.006597	<.01
Wednesday * Summer	− 2.758180	1.800220	− 1.523	0.125852	N.S.
Friday * Summer	− 4.491005	2.170668	− 2.069	0.038845	<.05
Tuesday * Fall	− 4.747532	1.754273	− 2.706	0.006937	<.01
Thursday * Fall	− 4.330716	1.751428	− 2.473	0.013600	<.05
Saturday * Winter	− 4.727871	2.171958	− 2.177	0.029765	<.05
Summer vacation week 1 + 2	− 7.220153	1.869252	− 3.863	0.000120	<.001
Summer vacation week 3 + 4	− 9.962722	1.862460	− 5.349	1.13e−07	<.001
Friday * Vacation	− 6.502580	2.961668	− 2.196	0.028385	<.05
Monday * Holiday	− 23.301127	3.296739	− 7.068	3.22e−12	<.001
Wednesday * Holiday	− 23.211391	6.806075	− 3.410	0.000682	<.001
Thursday * Holiday	− 25.870551	5.598786	− 4.621	4.40e−06	<.001
Friday * Holiday	− 26.937684	9.743801	− 2.765	0.005820	<.01
Time trend	0.010184	0.001397	7.292	6.87e−13	<.001
Sunday * Time trend	− 0.006243	0.003389	− 1.842	0.065813	N.S.
Summer vacation * time trend	− 0.013159	0.002659	− 4.948	8.99e−07	<.001
Carnival * Time trend	0.026781	0.008749	3.061	0.002274	<.01
Summer * Minimum wind speed Kaathoven	1.227642	0.422316	2.907	0.003742	<.01
Summer * Minimum wind speed Drunen	− 1.104485	0.439693	− 2.512	0.012186	<.05
Maximum temperature Drunen	0.033942	0.009225	3.680	0.000248	<.001
Winter * Maximum temperature Kaathoven	− 0.072152	0.023500	− 3.070	0.002205	<.01
Radiation Drunen	0.003902	0.001086	3.592	0.000347	<.001
Maximum pressure Kaathoven	− 0.014939	0.004470	− 3.342	0.000866	<.001
Maximum humidity Drunen	− 0.244718	0.099745	− 2.453	0.014345	<.05
Winter * Mean humidity Kaathoven	1.056103	0.460616	2.293	0.022096	<.05
Winter * Mean humidity Drunen	− 0.942567	0.465086	− 2.072	0.043002	<.05
Cloudiness Kaathoven	4.299755	1.479477	2.906	0.003750	<.01
Cloudiness Drunen	− 3.950170	1.452272	− 2.720	0.006658	<.01
Minimum visibility Kaathoven	− 0.028843	0.011739	− 2.457	0.014203	<.05
Fall * Minimum visibility Drunen	0.060823	0.016224	3.749	0.000189	<.001
Fall * Maximum visibility Drunen	− 0.011680	0.004764	− 2.451	0.014422	<.05
Precipitation duration Kaathoven	− 0.229262	0.101833	− 2.939	0.003382	<.01
Precipitation duration Drunen	0.349550	0.102416	3.413	0.000672	<.001
Summer * Precipitation Kaathoven (precipitation stations)	− 0.040857	0.023192	− 1.762	0.078436	N.S.
Summer * Precipitation Duration Drunen	− 0.127771	0.043480	− 2.939	0.003384	<.01
Summer vacation * Precipitation Kaathoven (precipitation stations)	0.138976	0.046988	2.958	0.003183	<.01
Summer vacation * Maximum Precipitation Drunen	− 0.105203	0.046440	− 2.265	0.023736	<.05
Hours of fog	1.254639	0.292019	4.296	1.93e−05	<.001
Spring * Hours of fog Drunen	− 0.708256	0.492974	− 1.437	0.151162	N.S.
Winter * Hours of fog Kaathoven	− 2.742514	0.928981	− 2.952	0.003240	<.01
Winter * Hours of fog Drunen	1.726884	0.951881	1.814	0.069993	N.S.
Winter * Hours of snow	− 1.150906	0.375299	− 3.067	0.002232	<.01
Residual standard error	9.439 on 872 degrees of freedom				
Multiple R-squared	0.5587				
Adjusted R-squared	0.5344				
F-statistic	23 on 48 and 872 degrees of freedom, *P*-value: < 2.2e−16				

Dataset B is based on calendar variables plus weather predictions at Kaathoven and Drunen; * = interaction; *N.S.* not significant

**Table 3:** Stepwise variable selection on dataset C

Variable	Coefficient	Standard error	Significance level
Intercept	308.6042	44.3327	<.01
Monday	12.5382	1.0441	<.01
Friday	9.718	1.0134	<.01
Saturday	− 8.5790	1.0223	<.01
Sunday	− 11.0413	1.0386	<.01
Winter	4.6875	1.0734	<.01
Time trend (daily)	0.0066	0.0013	<.01
Carnival	8.1048*	4.2351	N.S.
Holiday	− 14.0091	3.2413	<.01
Monday * Holiday	− 9.2721	4.7393	N.S.
Sunday * Holiday	13.9759	5.0697	<.01
Summer vacation (week 1 + 2)	− 12.1128	1.5751	<.01
Summer vacation (week 3 + 4)	− 16.4741	1.6734	<.01
Summer vacation (week 5 + 6)	− 6.4804	1.6781	<.01
Max temperature (in 0.1°C)	0.0196	0.0084	<.05
Global radiation (in J/cm^2^)	0.0038	0.0008	<.01
Max pressure (in 0.1 hPa)	− 0.0210	0.0043	<.01
Max visibility (in 100m)	− 0.0100	0.0044	<.05
Max humidity (in %)	− 0.1357	0.0726	N.S.
Snow (in hours)	− 0.7926	0.3286	N.S.
Fog (in hours)	0.4101	0.1583	<.01
Storm (in hours)	− 0.8429	0.4619	N.S.
Observations	921		
Multiple R-squared	0.4858		
Adjusted R-squared	0.4738		

Dataset C is based on calendar variables plus weather predictions at the Jeroen Bosch Hospital (the refined set of predictors containing the best predictors of datasets A and B); * = interaction; *N.S.* not significant

**Table 4:** Calendar-only model using all predictors from dataset D

Variable	Coefficient	Standard error	Significance level
Intercept	86.8072	1.0653	<.01
Monday	12.3425	1.0801	<.01
Friday	9.3426	1.0485	<.01
Saturday	− 8.8611	1.0513	<.01
Sunday	− 10.9487	1.0748	<.01
Summer	1,9174	1.2012	N.S.
Fall	− 3.2428	1.0004	<.01
Winter	− 1.5169	1.0585	N.S.
Time trend (daily)	0.007	0.0013	<.01
Carnival	9.632	4.3362	N.S.
Holiday	− 14.2356	3.3540	<.01
Monday * Holiday	− 9.2185	4.9002	N.S.
Sunday * Holiday	14.2801	5.2446	<.01
Summer vacation (week 1 + 2)	− 11.9859	1.7660	<.01
Summer vacation (week 3 + 4)	− 16.5986	1.8521	<.01
Summer vacation (week 5 + 6)	− 7.2858	1.811	<.01
Observations	921		
Multiple R-squared	0.4425		
Adjusted R-squared	0.4333		

Dataset D is based on calendar-based predictors selected from dataset C plus three season indicators (dropped spring to avoid multicollinearity); * = interaction; *N.S*. not significant

**Table 5:** Effects of calendar events on the number of predicted ER visits

Event	ER visits
Tuesday–Thursday (baseline)	89.2
Monday	+ 13.8%
Friday	+ 10.5%
Saturday	− 9.9%
Sunday	− 12.3%
Carnival	+ 10.8%
Holiday (Monday)	− 26.3%
Holiday (Tuesday–Saturday)	− 16.0%
Summer vacation (week 1 + 2)	− 13.4%
Summer vacation (week 3 + 4)	− 18.6%
Summer vacation (week 5 + 6)	− 8.2%
Time trend (per year)	+ 3.2%

**Table 6:** Prediction performance of the developed models

Model	MAPE
Empty model	12.198%
First set of predictors	9.893%
Second set of predictors	9.536%
Refined set of predictors	8.684%
Calendar-only model	8.718%

MAPE was measured using the mean average prediction error (average percentage the test predictions are off compared to the true number of daily ED visits); *MAPE* mean absolute percentage error

## Discussion

Our study shows that adding weather variables did not substantially increase the performance of a linear model based on calendar variables in predicting the daily number of ED visits in a retrospective setting (MAPE 8.718% vs. 8.684%). Of course, the intended value of such a model is to predict future daily numbers of ED visits, implicating that weather forecasts instead of retrospective weather data would have to be used. The uncertainties inherent to weather forecasts would increase the error of the model that includes weather variables. Therefore, we conclude that a model based on calendar variables would be most suited for hospital EDs that are comparable to ours. Previous research work confirming that calendar variables have greater predictive value on the number of ED visits than weather variables can be found in the systematic review conducted by Wargon et al. and in a study of Marcilio et al. [2, 11].

A constant, moderate number of predicted ED visits were found for regular Tuesdays, Wednesdays, and Thursdays with an increased number of predicted ED visits during regular Mondays and Fridays, and a decrease during regular Saturdays and Sundays. A previous study in a tertiary hospital in Brazil and a study in four academic hospitals in France also showed a decrease in the number of ED visits at weekends [2, 20], and in the latter, also an increase in the number of ED visits at Mondays was reported [20]. This was reported earlier as the weekly cycle [11]. In our study, holidays that fall on Monday through Saturday and summer vacation showed a decrease in predicted ED visits. A decrease in the number of ED visits in August was also reported in the French study [20]; however, this pattern was not found in Brazil [2]. The number of ED visits in the Jeroen Bosch Hospital showed an upwards trend, with an increase of 3.2% every year, from a mean of approximately 85 ED visits each day in 2016 to an approximate of 103 predicted ED visits each day in 2022. This yearly increase in the number of ED visits is similar to the findings in the French hospitals [20].

Our study has several strengths and limitations. A strength of this study is the use of a linear model. More complex methods such as the (seasonal) autoregressive integrated moving average ((S)ARIMA), which is a time series model, are sometimes favored; however, we used a linear model as these are easiest to understand for non-statisticians [11, 20]. Marcilio et al. compared linear models with a time series model and found that the linear models were slightly superior to the time series model [2]. Wargon et al. also described a linear model as the superior method [20]. However, Whitt and Zhang concluded that a time series model outperformed a linear model [21]. Another strength of this study is the use of the MAPE as a measurement of fit. The MAPE is similar to the mean squared error (MSE) that is more commonly used in general. However, the MAPE is used more often in this context as it yields a more intuitive interpretation of the error and thus allows for comparison with models made in similar works. The MAPE of approximately 8.7% in our linear model shows our model is accurate and comparable to several other studies showing the MAPE for linear models as well as time series models ranging between 4.2 and 14.4% [2, 11, 12, 21]. A limitation of our study is that we did not include our most recent data because of the disrupted situation caused by the COVID-19 pandemic. We presume this situation will stabilize in the future, but recalibration with data encompassing the post-COVID-19 situation will probably be useful. A second limitation is the small geographic area studied. It was therefore not possible to investigate cultural differences or population’s climatic adaptations. The similarity between the days with a decreased number of ED visits is that all are work-free days, often characterized by family visits. As the Netherlands have a temperate climate, our weather-related findings are potentially valid for other geographic areas with a temperate climate. A third limitation of our study is the need for interpolation of the weather data, as interpolation is an estimate. However, by using data from all weather stations in the greater Jeroen Bosch Hospital area, and by comparing two models with weather predictions at the Jeroen Bosch Hospital and at the periphery of the Jeroen Bosch Hospital’s catchment area (Kaathoven and Drunen), we think our estimate is quite robust.

## Conclusion

As far as we know, this is the first model predicting ED visits using calendar and weather variables in the Netherlands. It has similar performance as prediction models described in the literature so far. In conclusion, our calendar based linear model is useful to predict the number of ED visits for EDs of the same size and in a similar region as the Jeroen Bosch Hospital. However, as shown in Fig. 1, the variability in ED visits is considerable. Therefore, when using this model in practice, one should always anticipate the possibility of unforeseen spikes and dips in ED visits that are not shown by the model.

## Supplementary Information

**Additional file 1.** List of calendar variables.

**Additional file 2.** List of weather variables.

**Additional file 3.** Details interpolation parameter estimation.

**Additional file 4.** List of variables in the refined set of predictors.

## Data Availability

The hospital dataset used during the current study are available from the corresponding author on reasonable request. The datasets containing national weather data are publicly available from the KNMI website.

## Abbreviations

AIC: Akaike Information Criterion
ED: Emergency department
MAPE: Mean absolute percentage error
MSE: Mean squared error
KNMI: Dutch National Meteorological Institute
(S)ARIMA: (Seasonal) Autoregressive integrated moving average

## References

[1] Nederlandse Zorgautoriteit (NZa). Update cijfers acute zorg 2019 - Nederlandse Zorgautoriteit [Internet]. 2020 [cited 2020 Oct 6]. Availab le from: https://puc.overheid.nl/nza/doc/PUC_301126_22/1/

[2] Marcilio I, Hajat S, Gouveia N (2013). Forecasting daily emergency department visits using calendar variables and ambient temperature readings. Acad Emerg Med.

[3] Burns K, Chernyak V, Scheinfeld MH (2016). Emergency department imaging: are weather and calendar factors associated with imaging volume?. Clin Radiol.

[4] Afilal M, Yalaoui F, Dugardin F, Amodeo L, Laplanche D, Blua P (2016). Forecasting the emergency department patients flow. J Med Syst.

[5] Affleck A, Parks P, Drummond A, Rowe BH, Ovens HJ (2013). Emergency department overcrowding and access block. CJEM..

[6] Access to acute care in the setting of emergency department overcrowding. CJEM. 2003;5(2):81–6. 10.1017/s1481803500008204. 10.1017/s1481803500008204 17475096

[7] Hoot NR, Aronsky D (2008). Systematic review of emergency department crowding: causes, effects, and solutions. Ann Emerg Med.

[8] Morley C, Unwin M, Peterson GM, Stankovich J, Kinsman L. Emergency department crowding: a systematic review of causes, consequences and solutions. PLoS One. 2018. Vol. 13 1–42 p. 10.1371/journal.pone.0203316 PMC6117060 30161242

[9] Pham JC, Seth Trueger N, Hilton J, Khare RK, Smith JP, Bernstein SL (2011). Interventions to improve patient-centered care during times of emergency department crowding. Acad Emerg Med.

[10] Gul M, Celik E (2020). An exhaustive review and analysis on applications of statistical forecasting in hospital emergency departments. Heal Syst.

[11] Wargon M, Guidet B, Hoang TD, Hejblum G (2009). A systematic review of models for forecasting the number of emergency department visits. Emerg Med J.

[12] Kam HJ, Sung JO, Park RW (2010). Prediction of daily patient numbers for a regional emergency medical center using time series analysis. Healthc Inform Res.

[13] Chen TH, Du XL, Chan W, Zhang K (2019). Impacts of cold weather on emergency hospital admission in Texas, 2004–2013. Environ Res.

[14] Medina-Ramón M, Schwartz J (2007). Temperature, temperature extremes, and mortality: a study of acclimatisation and effect modification in 50 US cities. Occup Environ Med.

[15] Curriero FC, Heiner KS, Samet JM, Zeger SL, Strug L, Patz JA (2002). Temperature and mortality in 11 cities of the eastern United States. Am J Epidemiol.

[16] Koninklijk Nederlands Meteorologisch Instituut (KNMI). Klimatologie Daggegevens van het weer in Nederland [Internet]. 2020 [cited 2020 Nov 9]. Available from: http://projects.knmi.nl/klimatologie/daggegevens/selectie.cgi

[17] Koninklijk Nederlands Meteorologisch Instituut (KNMI). Klimatologie Uurgegevens van het weer in Nederland [Internet]. 2020 [cited 2020 Nov 9]. Available from: http://projects.knmi.nl/klimatologie/uurgegevens/selectie.cgi

[18] Koninklijk Nederlands Meteorologisch Instituut (KNMI). Klimaatdata en –advies Download tijdreeksen van 325 KNMI-neerslagstations [Internet]. 2020 [cited 2020 Nov 9]. Available from: http://projects.knmi.nl/klimatologie/monv/reeksen/select_rr.html

[19] Shepard D. A two-dimensional interpolation function for irregularly-spaced data. Proc 1968 23rd ACM Natl Conf ACM 1968. 1968;517–24.

[20] Wargon M, Casalino E, Guidet B (2010). From model to forecasting: a multicenter study in emergency departments. Acad Emerg Med.

[21] Whitt W, Zhang X (2019). Forecasting arrivals and occupancy levels in an emergency department. Oper Res Heal Care.

