# Supplementary material for: Predicting emergency department visits in a large teaching hospital
Source: Int J Emerg Med. 2021 Jun 12;14:34. doi: 10.1186/s12245-021-00357-6 (PMC8196936; doi:10.1186/s12245-021-00357-6)
Supplement: Supplementary file 1 — Additional file 1. List of calendar variables. [file 12245_2021_357_MOESM1_ESM.docx]

**Additional file 1: List of calendar variables**

- **Day of the week** described using 7 indicator variables, one for every day of the week
- **Month** described using 12 indicator variables, one for every month
- **Summer vacation** described using three indicator variables for the first, middle and last two weeks of the summer vacation in the south of The Netherlands respectively
- **School vacations** all non-summer school vacations in the south of The Netherlands described using one indicator variable (Carnival vacation, May vacation, Fall vacation and Christmas break)
- **Holiday** all Dutch school holidays described using one indicator variable (New Year's day, Easter, King’s Day, Ascension Day, Pentecost, Saint Nicholas, Christmas, and New Year's eve)
- **Carnival** the Saturday, Sunday and Monday of Dutch carnival described using one indicator variable
- **Time trend** described using a daily time index variable
