# Supplementary material for: Predicting emergency department visits in a large teaching hospital
Source: Int J Emerg Med. 2021 Jun 12;14:34. doi: 10.1186/s12245-021-00357-6 (PMC8196936; doi:10.1186/s12245-021-00357-6)
Supplement: Supplementary file 2 — Additional file 2. List of weather variables. [file 12245_2021_357_MOESM2_ESM.docx]

**Additional file 2: List of weather variables**

***Automatic weather station variables measured on a daily basis***

**Wind speed**

- **Vector mean wind direction** measured in degrees
- **Vector mean wind speed** measured in 0.1 m/s
- **Daily mean wind speed** measured in 0.1 m/s
- **Maximum hourly mean wind speed** measured in 0.1 m/s
- **Minimum hourly mean wind speed** measured in 0.1 m/s
- **Maximum wind gust** measured in 0.1 m/s

**Temperature**

- **Mean temperature** measured in 0.1 degrees Celsius (at a height of 1.5m)
- **Minimum temperature** measured in 0.1 degrees Celsius (at a height of 1.5m)
- **Minimum temperature at a height of 10 cm** measured in 0.1 degrees Celsius
- **Maximum temperature** measured in 0.1 degrees Celsius (at a height of 1.5m)

**Radiation**

- **Sunshine duration** measured in 0.1 hours (less than 0.05 hours of daily sunshine is rounded to 0 hours)
- **Percentage of maximum potential sunshine duration** measured as percentage (measured sunshine duration as percentage of maximum possible sunshine duration)
- **Global radiation** measured is J/cm^2^
- **Potential evaporation** measured in 0.1 mm (amount of evaporation assuming enough water is present)

**Pressure**

- **Daily mean sea level pressure** measured in 0.1 hPa (based on 24 hourly values)
- **Maximum hourly sea level pressure** measured in 0.1 hPa
- **Minimum hourly sea level pressure** measured in 0.1 hPa

**Visibility**

- **Minimum visibility** measured in 100m (rounded down)
- **Maximum visibility** measured in 100m (rounded down)

**Cloudiness**

- **Mean daily cloud cover** measured in octants (cloud measurement from 0, clear sky, to 8, completely cloudy)

**Humidity**

- **Daily mean relative atmospheric humidity** measured as percentage (amount of water in the air over the maximum amount of water the air can hold at given circumstances)
- **Maximum relative atmospheric humidity** measured as percentage
- **Minimum relative atmospheric humidity** measured as percentage

**Precipitation**

- **Precipitation duration** measured in 0.1 hours
- **Precipitation amount** measured in 0.05 mm (less than 0.05 mm is rounded to 0.05 mm)
- **Maximum hourly precipitation amount** measured in 0.05 mm (less than 0.05 mm is rounded to 0.05 mm)

***Automatic weather station variables measured on an hourly basis***

**Wind speed**

- **Wind direction during the last 10 minutes** measured in degrees
- **Mean wind speed** measured in 0.1 m/s (measured over the whole hour)
- **Mean wind speed during the last 10 minutes** measured in 0.1 m/s
- **Maximum wind gust** measured in 0.1 m/s

**Temperature**

- **Temperature** measured in 0.1 degrees Celsius (at a height of 1.5m)
- **Minimum temperature at a height of 10 cm** measured in 0.1 degrees Celsius (measured on a 6-hourly basis)
- **Dew point temperature** measured in 0.1 degrees Celsius (temperature at which water will condense, measured at a height of 1.5m)

**Radiation**

- **Sunshine duration** measured in 0.1 hours (less than 0.05 hours is rounded to 0 hours)
- **Global radiation** measured is J/cm^2^

**Pressure**

- **Pressure** measured in 0.1 hPa

**Visibility**

- **Visibility** measured in 100m (rounded down)

**Cloudiness**

- **Cloudiness** measured in octants

**Humidity**

- **Relative atmospheric humidity** measured as percentage (at a height of 1.5m)

**Precipitation**

- **Precipitation duration** measured in 0.1 hours
- **Maximum hourly precipitation amount** measured in 0.05 mm (less than 0.05 mm is rounded to 0.05 mm)
- **Rain** binary variable indicating if rain was observed
- **Snow** binary variable indicating if snow was observed

**Special weather conditions**

- **Fog** binary variable indicating if fog was observed
- **Storm** binary variable indicating if a storm was observed
- **Glazed frost** binary variable indicating if glazed frost was observed

***Precipitation stations variables measured on daily basis***

- **Precipitation** measured in 0.1 mm (measurement is based on precipitation between 8 am last day and 8 am current day)
- **Snow** measured in cm (broken snow cover less than 1 cm thick is rounded to 1 cm, broken snow cover more than 1 cm thick is rounded to 2 cm, snow dunes are rounded to 4 cm, all measurements are conducted at 8 am)
