# Supplementary material for: Predicting emergency department visits in a large teaching hospital
Source: Int J Emerg Med. 2021 Jun 12;14:34. doi: 10.1186/s12245-021-00357-6 (PMC8196936; doi:10.1186/s12245-021-00357-6)
Supplement: Supplementary file 3 — Additional file 3. Details interpolation parameter estimation. [file 12245_2021_357_MOESM3_ESM.docx]

**Additional file 3: Details interpolation parameter estimation**

The parameter estimation for weather variables extracted from automatic weather stations was done by making numerous weather predictions for the weather at weather station Herwijnen, the only automatic weather station within the catchment area of the Jeroen Bosch Hospital, during the period from 2011 through March 2020. These weather predictions were created by applying inverse distance weighting using a large range of values for the interpolation parameter on weather observations from all automatic weather stations within 50km of Herwijnen: Volkel, Eindhoven, Gilze Rijen, Cabauw, De Bilt, and Rotterdam. The parameter value whose predictions yielded the lowest sum of squared residuals were selected for all other predictions of this weather variables. The parameters can differ between weather variables.

The parameter estimation of the two weather variables extracted from the precipitation stations was done by expanding on the previous methodology. The quality of the predictions were once again assessed using the sum of squared residuals, however here the sum of squared residuals followed from predictions at 9 precipitation stations within the catchment area of the Jeroen Bosch Hospital: Ammerzoden, Andel, Boxtel, Dinther, Giersbergen, Herwijnen, Nuland, Oss, and Zaltbommel, using the 29 other precipitation stations within a 37km range of the Jeroen Bosch Hospital (this includes the other 8 precipitation stations in the catchment area). This 37km range was selected as it yielded the lowest total sum of squared residuals out of all possible ranges.
