# Supplementary material for: Predicting emergency department visits in a large teaching hospital
Source: Int J Emerg Med. 2021 Jun 12;14:34. doi: 10.1186/s12245-021-00357-6 (PMC8196936; doi:10.1186/s12245-021-00357-6)
Supplement: Supplementary file 4 — Additional file 4. List of variables in the refined set of predictors. [file 12245_2021_357_MOESM4_ESM.docx]

**Additional file 4: List of variables in the refined set of predictors**

**Calendar variables**

- **Day of the week** described using six indicator variables, one for every day of the week except Tuesday
- **Season** described using three indicator variables, one for every season except spring
- **Summer vacation** described using three indicator variables for the first, middle and last two weeks of the summer vacation in the south of The Netherlands respectively
- **School vacations** all non-summer school vacations in the south of the The Netherlands described using one indicator variable (Carnival vacation, May vacation, Fall vacation and Christmas break)
- **Holiday** all Dutch school holidays described using one indicator variable (New Year’s Day, Easter, King’s Day, Ascension Day, Pentecost, Saint Nicholas, Christmas and New Year’s Eve)
- **Carnival** the Saturday, Sunday and Monday of Dutch carnival described using one indicator variable
- **Time trend** described using a daily time index variable

**Weather variables**

For the following weather variables, the weather prediction at the Jeroen Bosch Hospital and the absolute difference between the weather predictions at Kaathoven and Drunen are included in the refined set of predictors:

- **Minimum wind speed**
- **Maximum temperature**
- **Global radiation**
- **Maximum pressure**
- **Maximum visibility**
- **Cloudiness**
- **Maximum humidity**
- **Precipitation duration**
- **Daily hours of snow**
- **Daily hours of fog**
- **Daily hours of storm**
- **Daily hours of glazed frost**

**Interaction variables**

- **Day of the week and Holiday** described using six indicator variables, one for every day of the week interaction except Tuesday
- **Friday and school vacations** described using one interaction variable
